# Enrichment of Cellulose Acetate Nanofibrous Scaffolds with Retinyl Palmitate and Clove Essential Oil for Wound Healing Applications

**DOI:** 10.1021/acsomega.2c06881

**Published:** 2023-02-01

**Authors:** Aysen Akturk

**Affiliations:** Department of Chemical Engineering, Istanbul Technical University, Istanbul34469, Turkey

## Abstract

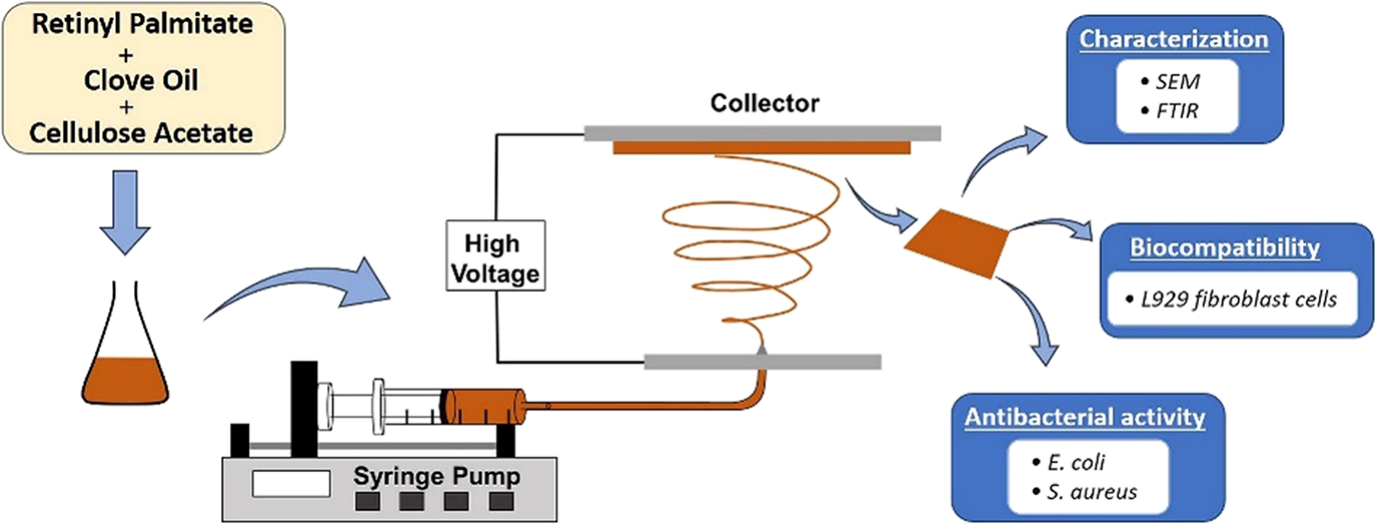

The use of biocompatible materials and fabrication methods is of particular importance in the development of wound dressings. Cellulose acetate (CA) has excellent properties for wound dressing applications, but it is insufficient for the wound healing process due to its lack of bioactive and antibacterial properties. In this study, CA was electrospun with retinyl palmitate (RP) and clove essential oil (CLV) to fabricate a novel antibacterial and antioxidant biomaterial. The effects of RP and CLV incorporation on the surface morphology, fiber diameter, antioxidant activity, antibacterial activity, cell viability, and release behavior of the fabricated CA mats were investigated. In light of these studies, it was determined that the nanofiber mat, fabricated with a 15% w/v CA polymer concentration, a 1% w/w RP ratio, and a 5% w/w CLV ratio, was biocompatible with L929 fibroblast cells with antibacterial and antioxidant properties. Overall, results showed that this nanofiber offers promise for use as a wound dressing.

## 1 Introduction

The development of scaffolds that are ideal for the regeneration of injured tissues while also allowing for the restoration of their biological functions has long been a major goal in the field of tissue engineering research. Electrospinning is one of the methods that has attracted significant attention as a potential candidate for developing such scaffolds.^1^ Due to their fibrous structure to imitate the extracellular matrix (ECM) architecture of human skin, which facilitates cell adhesion and proliferation and promotes the development of new tissue, electrospun nanofibrous mats with their controllable pore structure and continuous uniformity have emerged as promising solutions for wound healing management.^1−5^ Their permeability to moisture and air allows for the proper removal of surplus bodily fluid from the wound region to prevent infection and maintain a moist environment.^1−4^

Additionally, the outstanding mechanical characteristics of electrospun membranes ensure that they can withstand mechanical stresses during handling and regeneration. Also, electrospun nanofibers may operate as drug delivery systems with efficient/sustained drug release characteristics, reducing the frequency of topical treatments.^4^ Numerous therapeutic agents (natural remedies, anti-inflammatory drugs, antibiotics, antioxidants, silver-based materials, vitamins, minerals, and growth factors) have been added to improve the biological performance of electrospun fibers, allowing for the easy fabrication of multifunctional dressings.^2−4,6,7^

Cellulose acetate (CA) is the acetate ester of cellulose, which is the most prevalent biopolymer in nature. CA is a biocompatible and biodegradable material with high mechanical performance.^8^ It holds great potential for controlled release applications because of its capability to spin into fibers and dissolve in a variety of solvents. Thus, the electrospinning of CA into membranes with varied fiber diameter, porosity, and thickness, is performed more successfully than that of natural cellulose.^9^ Despite their numerous advantages, nanofibrous scaffolds, produced from only CA, show a lack of bioactivity and antibacterial activity for successful wound healing.^10^

Vitamin A is important in many metabolic processes, including cellular differentiation, growth, immunity, epithelial integrity, collagen synthesis, and angiogenesis; thus, it has potential applications in wound healing.^11−13^ Taking its stability into consideration, retinyl palmitate (RP), a derivative of vitamin A, exhibits greater stability than vitamin A.^7,14^ RP is the ester of palmitic acid and retinol, which is the major active form of vitamin A. RP is converted into retinol under physiological conditions, and it is used for the treatment of skin diseases.^15^ It affects epithelization in dry and rough skin and abnormal keratinization.^7,16^ Therefore, CA nanofiber mats endowed with RP have a potential to enhance wound healing capability.

Previous studies demonstrated that the addition of antibiotics, antiviral medicines, titanium dioxide, zinc oxide, and silver compounds might increase the antibacterial activity of cellulose acetate nanofibers.^17^ However, due to the nature of the materials that are utilized in these applications, certain issues may arise. Antimicrobial resistance of biomaterials endowed with antibiotics represents a significant challenge to public health. UV applications are required for the activation of titanium dioxide and zinc oxide. The preparation of metal nanoparticles calls for sophisticated processes.^17,18^ In addition, there exists the possibility of the accumulation of metallic nanoparticles in human organs.^19^ Thus, essential oils (EOs) are emerging as alternative antibacterial agents for sustainable and biocompatible applications.^17^ Due to their low toxicity and broad-spectrum antimicrobial activity, they demonstrate promising effects against pathogenic bacteria and micro-organisms in wound dressing applications.^20^ Several essential oils, such as *Zataria multiflora*,^10^ lemon myrtle,^9^ rosemary,^21^ oregano,^21^ cinnamon,^22^ lemongrass,^22^ peppermint,^22^ and sambong oil,^23^ were studied to fabricate antibacterial CA nanofiber mats. With the advantage of their cellulose origin, CA-based nanofibers retain the EOs for a long time and are considered as effective materials in wound dressings enriched with EOs.^21^

Clove essential oil (CLV), a GRAS (generally recognized as safe), has been used medicinally for centuries for its therapeutic effects. It is extracted from the aromatic flower buds of *Eugenia caryophyllata* and *E. aromaticum*.^24,25^ Eugenol (∼80%) is the main volatile component of CLV, responsible for its powerful antioxidant and antimicrobial activities.^24,26^ The optimal dosage of CLV enhanced cell viability.^24^ Its practical use is questioned due to its volatility, low viscosity, and sensitivity to oxygen, heat, and light.^24,27,28^ Different scaffold preparation methods have been used to develop polymer carriers for CLV, such as cast films, nanoparticles, and, more recently, electrospun fiber mats to improve the applicability of CLV.^24^

This study aimed to investigate the potential of CA nanofibers combined with RP and CLV as electrospun bioactive wound dressing materials. Morphological and chemical composition characterizations, biocompatibility, antibacterial activity, antioxidant capacity, and controlled release behaviors of RP- and CLV-loaded CA nanofiber mats were discussed. The influence of RP addition and CLV addition at various concentrations was examined, and a composition with the ability to act as a wound dressing was determined. This research was conducted for the first time using a wound dressing material obtained by incorporating RP and CLV into CA nanofibers by an electrospinning technique.

## 2 Results and Discussion

### 2.1 SEM Analysis

**Figure 1:**
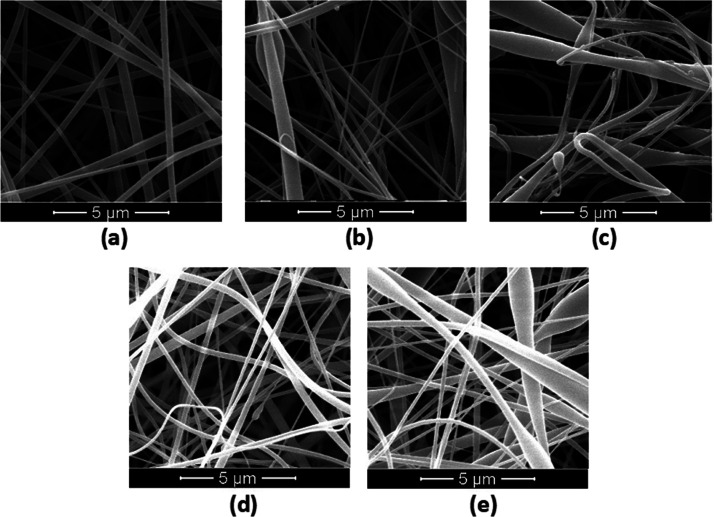
SEM images of CA (a), CA/RP (b), CA/RP/5CLV (c), CA/RP/10CLV (d), and CA/RP/15CLV (e).

In this study, CLV and RP were used to develop electrospun CA fiber composites as carriers for active essential oils and vitamins. The effects of RP and CLV loading on the morphological appearance and size of as-spun CA nanofibers were studied. The morphologies of neat CA, CA/RP, and CA/RP nanofiber mats with varying CLV weight ratios to CA of 5, 10, and 15 wt/wt % (CA/RP/5CLV, CA/RP/10CLV, and CA/RP/15CLV) were characterized by SEM (Figure 1). Pure CA solution, 15% wt/v, was able to produce electrospun fibers in the sub-micrometric scale to record 350 ± 97 nm. However, spindle-like beads were observed with the encapsulation of RP in the structure. This result agrees with the literature.^7,11^ It has been reported that this could be attributed to the electrical conductivity of the solution. A decrease in the electrical conductivity caused insufficient stretching of the derived jet, producing nanofibers with thicker diameters, which prevented uniform fiber formation and led to bead defects.^11^ Moreover, the addition of vitamins increases the viscosity of the electrospinning solutions, and higher viscosities can also result in the formation of beaded nanofibers.^7^ The average fiber diameters of the CA/RP, CA/RP/5CLV, CA/RP/10CLV, and CA/RP/15CLV nanofiber mats were found as 308 ± 311, 300 ± 255, 314 ± 285, and 265 ± 225 nm. It can be observed that neat CA nanofibers had a uniform diameter distribution (lower standard deviation value). In contrast, CA/RP, CA/RP/5CLV, CA/RP/10CLV, and CA/RP/15CLV nanofibers exhibited a wide range of diameters with the incorporation of RP and CLV into the structure. However, there was not a significant variation in the diameters of the obtained nanofiber mats (*p* > 0.05). When compared to the three-dimensional collagen fibril network and the nanoscale range of the natural extracellular matrix (50–500 nm), it can be shown that the produced nanofibers are in the range that is acceptable for fibroblast adhesion and proliferation.^29,30^

### 2.2 Antibacterial Activity and Cytotoxicity of Membranes

The potential of the RP- and CLV-loaded CA fiber mats for use as wound dressing materials was investigated by determining their antibacterial activity and cytotoxicity.

**Figure 2:**
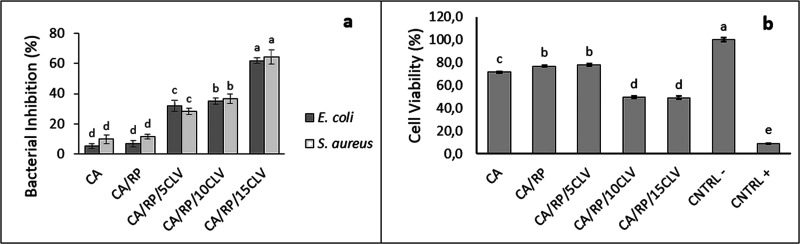
Antibacterial activity (a) and cell viability (b) of RP- and CLV-loaded CA nanofibers. *Bars sharing the same letter are not significantly different at *p* > 0.05, *n* = 3.

Antimicrobial tests were carried out on two bacteria (one Gram-positive, *Statphylococcus aureus*, and one Gram-negative, *Escherichia coli*). They are the most widely researched model micro-organisms for studying pathogenicity, resistance, the development of infectious processes, and biofilm formation. These opportunistic bacteria cause numerous community and hospital illnesses. One in three persons carries *S. aureus* in their nose or pharynx, making it a common cause of skin and wound infections. This strain is responsible for dangerous infections in critical care patients, where antibiotic-resistant bacteria (like MRSA: methicillin-resistant *S. aureus*) are often identified. The capability of *S. aureus* to form resistant biofilms on indwelling devices, medical surfaces, and open wounds restricts therapy options. The Gram-negative study model *E. coli* can lead to dangerous illnesses ranging from gastrointestinal and urinary tract infections.^21^ According to several studies, the antibacterial properties of essential oils come from their ability to disrupt the ion and solute transport processes that occur within bacteria. In addition, a number of studies have found that lipophilic EOs have the ability to pass through the membrane of bacteria, which ultimately results in the death of the cells.^9,31^ Phenolic compounds, which are components of essential oils, permeate the cell walls of bacteria and exert antibacterial action by blocking energy-producing enzymes or denaturing proteins in the cell wall, causing the cell wall barrier to be damaged.^23^ The antibacterial activity of CA, CA/RP, CA/RP/5CLV, CA/RP/10CLV, and CA/RP/15CLV nanofiber mats against both bacteria strains is shown in Figure 2a. When compared to CA and CA/RP nanofiber mats, the antibacterial activity of nanofiber mats containing CLV showed significant improvement. This improvement can be explained by the different amounts of the compounds responsible for the antimicrobial activity in this oil (primarily phenolic compounds).^26^ Eugenol is the primary constituent (∼80%) of CLV.^26,27,32^ It has a wide variety of biological activities, including those that are insecticidal, antifungal, anticarcinogenic, antiallergic, and antimutagenic. It also possesses antioxidant characteristics.^32^

In studies examining the effectiveness of membranes enriched with clove oil against *E. coli* and *S. aureus* bacteria, it is seen that these membranes are more effective against *S. aureus* than *E. coli* due to the different composition of their cell wall structures.^25,33^ However, similar to this study, there are studies that have similar effects of clove oil against both bacteria.^34−36^ This is thought to be based on the type of spice or herb used for extraction of essential oils, the type of the culture of the sample, and the characteristic of the film matrix.^36−39^

Another crucial aspect of materials in the biomedical field is their cytotoxic effects on healthy cells. Herein, the cytotoxicity of CA, CA/RP, CA/RP/5CLV, CA/RP/10CLV, and CA/RP/15CLV nanofibers toward the L929 cell line was assessed by cell viability after 48 h of incubation (Figure 2b). The ISO-10993-5 states that a cell vitality of more than 80% is nontoxic, the cell viability of between 80 and 60% is weakly toxic, the cell viability of between 60 and 40% is moderately toxic, and the cell viability of less than 40% is highly toxic.^23^ CA nanofibers showed weak toxicity with 71.5% cell viability. Similarly, Ullah et al. found that CA nanofibers showed weak toxicity than the control in their cytotoxic study.^23^ After adding RP, the cell viability increased slightly but exhibited weakly toxic behavior with 76.9% cell viability. It was seen that the cytotoxic properties of the nanofiber structure did not change and showed weak toxicity with 78% cell viability with the addition of 5% CLV. However, the contribution of 10 and 15% CLV makes the nanofiber structure moderately toxic, with 49.9 and 49.2% cell viability, respectively. Similar findings have been found in other studies, indicating that the CLV additive has a toxic impact when applied to electrospun nanofiber membranes at increasing concentrations.^20,40^ Since the cell viability was 78%, the toxicity level of CA/RP/5CLV fibers was found to be suitable for use as a wound dressing.^41^

Antibacterial and cytotoxic test findings were taken into consideration, and further studies were conducted with the CA/RP/5CLV nanofiber membrane, which is acceptable for wound dressing applications.

### 2.3 FTIR Results

**Figure 3:**
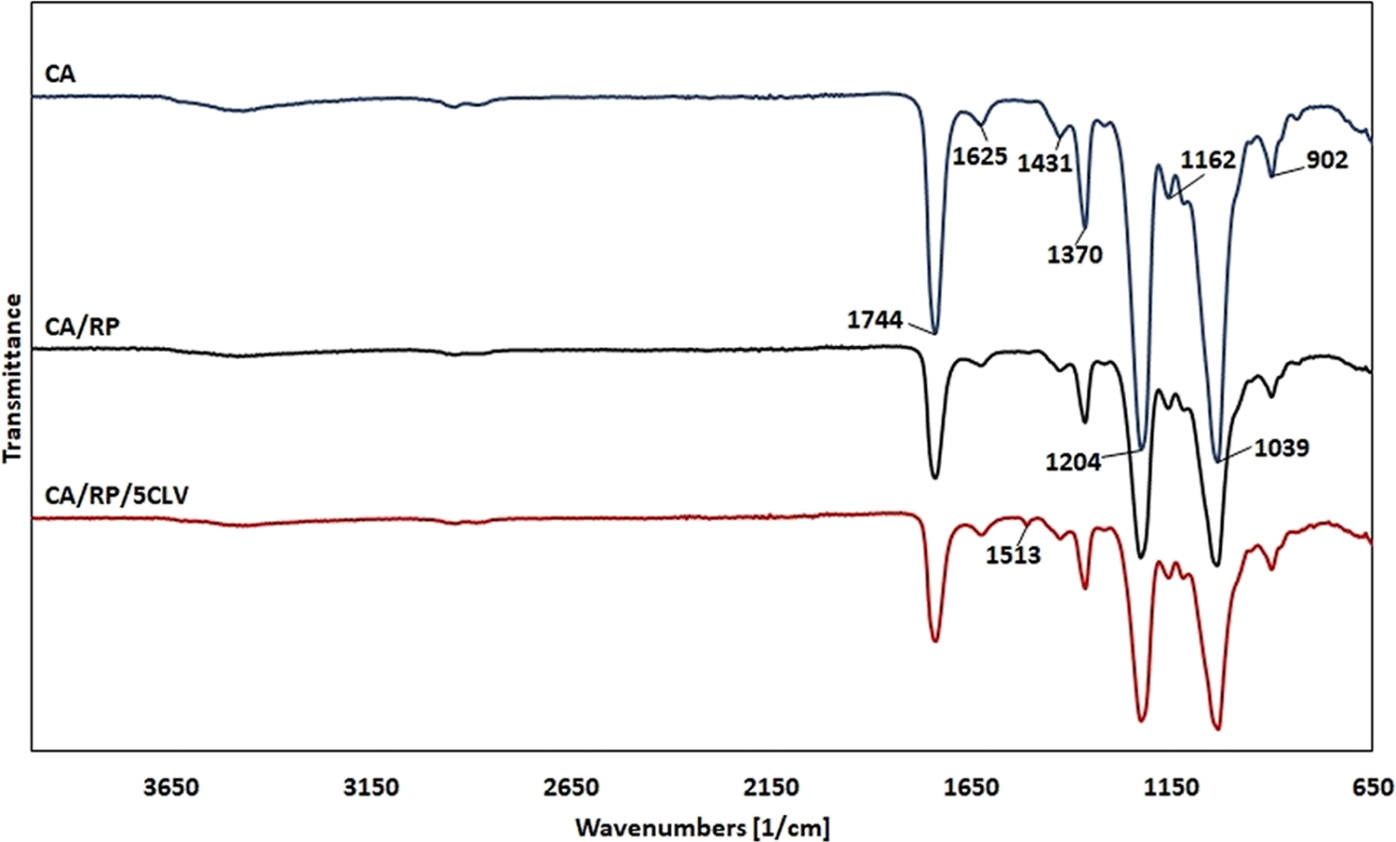
FTIR spectra of CA, CA/RP, and CA/RP/5CLV nanofiber membranes.

FTIR measurements were performed to demonstrate that CLV was successfully encapsulated within CA/RP/5CLV electrospun fibers (Figure 3). The FTIR spectra of CA electrospun nanofibers indicated the characteristic bands attributed to the acetate group. The stretching bands of carbonyl (C=O stretching) at 1744 cm^–1^, methyl bending at 1370 cm^–1^ (C–CH_3_ stretching), the alkoxyl stretch of the ester at 1204 cm^–1^ (C–O–C antisymmetric stretching ester group), and the C–O functional group in the absorption region of 1039 cm^–1^ were observed for the CA electrospun nanofibers.^9,10,18,42^ Glycosidic linkage of CA was observed at 1162 cm^–1^.^10^ Furthermore, the band at 1625 cm^–1^ can be linked with the presence of water molecules.^18^ When comparing the FTIR spectra for neat CA and CA/RP electrospun nanofibers, no shifts or flattening in the peaks was observed, suggesting no significant FTIR-sensitive chemical interactions between the RP and CA. According to the published data on CLV, peaks that are indicative of eugenol, which is the primary component of CLV, could be seen at 3522 cm^–1^ (O–H stretching), 1231 cm^–1^ (C–O bending), 1609 cm^–1^, 1512 cm^–1^, and 1430 cm^–1^ (C–C stretching vibrations in the phenyl ring).^43^ However, the overlapping of intense absorption peaks of CA made it difficult to detect the presence of CLV in spectra of CA/RP/5CLV. Nevertheless, a weak peak at 1513 cm^–1^ was found, which was assigned to the characteristic absorption peak of the phenyl ring of CLV.^24,28^ The C=C aromatic band of CLV observed as a small band in the CA/RP/5CLV nanofibers indicated that the CLV was successfully entrapped in the CA nanofibers after electrospinning.

### 2.4 In Vitro Release Behavior of RP and CLV

**Figure 4:**
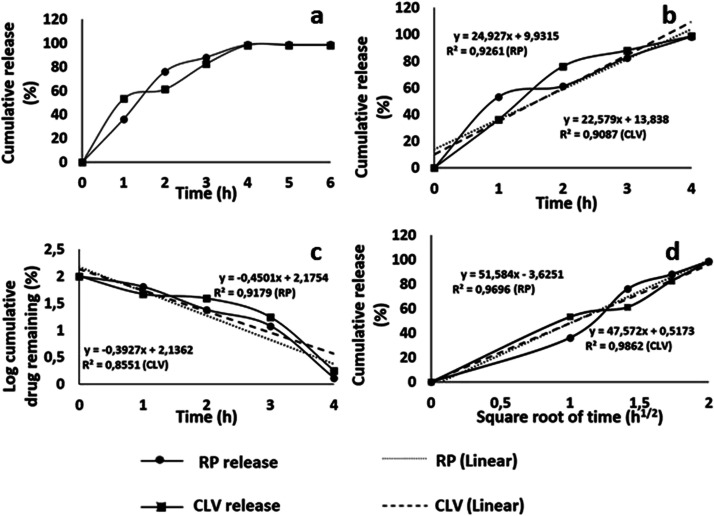
RP and CLV in vitro release profiles in PBS. Cumulative release behavior (a), zero-order kinetic model (b), first-order kinetic model (c), and Higuchi model (d).

Figure 4 shows the CLV and RP release profiles of the CA/RP/5CLV nanofiber mat. As seen in the drug release profile, the cumulative drug release increases linearly with time over a 4 h period (Figure 4a). The CLV and RP concentrations in the medium remained unchanged after 4 h. The results indicated that 98.22 and 98.70% of CLV and RP were released from the nanofiber mat within 4 h, respectively. The drug release behavior for the nanofibrous sample in 4 h was evaluated by various drug delivery release mechanisms, including the zero-order kinetic model, first-order kinetic model, and Higuchi model. It is clear that the Higuchi model describes the release of CLV and RP from the CA fibrous mats based on the highest correlation coefficient. This model states that drug release occurs by diffusion.^8^ In the Higuchi model, the release kinetics are controlled mainly by the dispersed phase diffusing out of the matrix, which leads to the dissolution of the matrix and the release of the dispersed phase.^44^ The Fick-type release mechanism of CLV and RP from the CA matrix is confirmed by the Higuchi model (Figure 4d).

### 2.5 Determination of Antioxidant Activity

During the inflammatory phase of wound healing, biologically active mediators will attract neutrophils, leukocytes, and monocytes to the wound site, where they will destroy bacteria and foreign debris via phagocytosis.^45^ This process will result in a sharp increase in free radicals such as a superoxide anion, hydrogen peroxide, and hydroxyl anion.^45,46^ Excessive levels of these free radicals alter the cellular oxidant/antioxidant equilibrium, induce enzyme inactivation, DNA damage, and lipid peroxidation, and delay the wound healing process.^45^ This damage can lead to cardiovascular diseases, diabetes, cancer, and faster aging.^47^ One key technique for enhancing wound healing is to reduce/scavenge free radicals surrounding wound sites by introducing antioxidant substances, therefore protecting cells or tissues from harm and accelerating wound healing.^45,48^ Various natural compounds, such as gum composites, essential oils, and volatile natural mixtures, have antioxidant properties. The DPPH radical scavenging activity test has been utilized to evaluate antioxidant agents as free radical scavengers or hydrogen donors.^48^

Eugenol, the primary phenolic component of CLV, has antioxidant properties.^49^ Antioxidant properties are also present in RP.^16^ In DPPH tests carried out with CA/RP/5CLV nanofibers at concentrations of 0.1, 0.2, and 0.5 mg/mL, values of 18.84, 47.67, and 51.24% radical inhibition were found, respectively. These results indicated that the investigated nanofiber exhibits radical scavenging activity. CLV and RP, known to have antioxidant effects, still have antioxidant properties even though the polymer solution was exposed to a high electrical potential during the electrospinning process. Similar results were also reported from the literature that the antioxidant activity of bioactive compounds was preserved after loading in the CA matrix and fabricating by electrospinning.^8,23,41,50^ According to the results of the DPPH test, the release of CLV and RP from the CA/RP/5CLV nanofiber may quench hydrogen peroxide-induced free radicals and protect the cells against oxidative stress, and this nanofiber has the potential to accelerate the in vivo wound healing process.^45,51^

## 3 Conclusions

In this work, cellulose acetate (CA) wound dressings were prepared by incorporating retinyl palmitate (RP), a vitamin A derivative, as a therapeutic wound healing agent and clove essential oil (CLV) as a natural antibacterial agent. These wound dressings were fabricated using the electrospinning technique, gaining interest in developing membranes for tissue engineering applications. Herein, a fixed concentration of RP (1% wt/wt with respect to CA) was added to the CA polymer solution for the fabrication of RP-blended nanofibrous membranes. CA/RP/CLV nanocomposite membranes with different concentrations of CLV (5, 10, and 15% wt/wt with respect to CA) were prepared. Morphological evaluation of scaffolds by using scanning electron microscopy showed that RP and CLV were integrated to the CA nanofibrous structure, and spindle-like beads were observed with the encapsulation of RP and CLV. The antimicrobial activity and cell proliferation study of membranes were studied using *E. coli* (Gram negative) and *S. aureus* (Gram positive), and L 929 fibroblasts as model strains and cell line, respectively. The results show that the CA nanofiber mat loaded with 5% wt/wt CLV and 1% wt/wt RP was found to be antibacterially effective as well as cytotoxically biocompatible. With this membrane, additional FTIR, in vitro release, and antioxidant capacity analyses were conducted. These findings revealed that RP and CLV were able to successfully participate in the CA nanofibrous membrane, and this membrane possessed antioxidant properties. It was found to be suitable for use as a wound dressing.

## 4 Materials and Methods

### 4.1 Materials

Cellulose acetate (CA: Mn = 30,000, Sigma Aldrich, USA) was used to fabricate pristine and composite nanofiber membranes. Clove oil (1.004 g/mL, containing 81.93% eugenol and 12.28% β-caryophyllene) was donated from Aromsa Besin Aroma ve Katkı Maddeleri Sanayi Ticaret A.Ş. (Kocaeli, Turkey). Retinyl palmitate (RP) and *N*,*N*-dimethylacetamide (DMAc puriss. p.a. 99.5%) were purchased from Sigma Aldrich. Acetone was obtained from Merck.

### 4.2 Fabrication of Pristine and CA/RP/CLV Membranes

The facile electrospinning process was carried out for the fabrication of pristine ultrafine CA and CA/RP/CLV membranes by using an electrospinning device (Nanospinner 24 Touch, Inovenso Co.). In a usual process, CA solution (15 wt %) was formulated by dissolving CA in DMAc:acetone (1:2 v/v) solution based on our preliminary studies. Various CLV weights (5–15%) were added to the polymer solution. Then, 1 wt % of RP was added to each CA/CLV polymer solution and stirred overnight at room temperature to get uniform CA/RP/CLV polymer solutions (CA, CA/RP, CA/RP/5CLV, CA/RP/10CLV, and CA/RP/15CLV). The amount of RP in the CA solutions was based on the amount of 1 wt % stated in the literature.^7^ The obtained solutions were electrospun separately. Briefly, polymer solutions were put into 5 mL syringes. A copper pin connected to a high voltage power supply was used, and an aluminum foil was covered around the collector. The applied voltage, tip-to-collector distance, and flow rate for electrospinning were 25 kV, 13 mm, and 1.5 mL·h^–1^, respectively. The electrospun mats were collected and stored in desiccators for further use.

### 4.3 Characterization of Membranes

The morphological appearance of the pristine CA and CA composite mats was observed by a scanning electron microscope (SEM, JSM-5410, Jeol) operated at 20 kV. Each sample was uniformly spread on carbon tape, and Pt coating was applied for 120 s onto the synthesized nanofibers prior to SEM observation. The functional groups of the pristine and composite fibers were analyzed by Fourier transform infrared spectroscopy (FTIR, Perkin Elmer Spectrum 100 model spectrometer) in transmittance mode in the mid-IR region (4000–650 cm^–1^).

### 4.4 Antibacterial Assessment

The optical density (OD) technique was used to evaluate antibacterial activity as described elsewhere. Both *E. coli* ATCC 25923 and *S. aureus* ATCC 25922 were cultured in 100 mL of nutrient broth (1 g/L meat extract, 5 g/L peptone from meat, 2 g/L yeast extract, and 5 g/L NaCl). A 10^4^ CFU ml^–1^ was prepared, and 10 μL was dispensed into 10 mL fresh nutrient broth solution. Fifty milligrams of the fibrous mat was added to tubes and incubated for 24 h. Tubes without fibrous mats were assumed to be controls. After 24 h, the absorbance of the nutrient broth solutions was measured at 600 nm using a UV–vis spectrophotometer (BioTek Synergy HT). The bacterial reduction was measured as a percentage using eq 1(23)
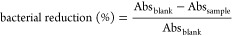
1

Here, Abs_blank_ is the absorbance of the empty tube, and Abs_sample_ is the absorbance of the tubes containing fiber samples. For bacterial reduction with the contact time, the absorbance of the sample tubes was taken after 24 h and compared with the absorbance of the empty tube. The percent reduction was calculated according to eq 1.

### 4.5 Cell Culture Study

A direct contact test between the materials and mouse fibroblast (L929) cells was used to investigate the cytotoxicity of the produced materials. In a typical procedure, cells were first cultured in an RPMI culture medium supplemented with 10% FBS. The culture medium was incubated under standard culture conditions (37 °C, 5% CO_2_, and 85% humidity). After 24 h, the cells were separated from the flask by the trypsin enzyme. Prepared specimens were sterilized under UV light exposure and placed in each 96-well plate at a density of 10^5^ cells per well. The cell culture medium without any nanofibrous mats was used as a negative control. Then, 1% phenol solution was used as the positive control. The plate was incubated at 37 °C for 48 h. The amount of living cells was determined by the MTT assay. After 48 h, MTT dye was added to each well of the 96-well plate. Eventually, dimethyl sulfoxide (DMSO) was added to each well, and optical densities were determined at 570 nm. Experiments were carried out in three repetitions.

### 4.6 In Vitro Release Study

The total immersion method into PBS (pH 7.4) containing 0.5%v/v Tween 80 was used to measure the release of RP and CLV oil from fibrous mats. The maximum absorption wavelengths of CLV oil in PBS (280 nm) and RP in PBS were determined using spectra over wavelengths ranging from 200 to 700 nm, which is consistent with other studies.^32,52^ The RP and CLV oil calibration curves were prepared in PBS using a UV–vis spectrophotometer (BioTek SynergyHT) at their maximum absorption wavelengths. Fibrous mats (50 mg) were immersed in PBS solution for 0–6 h at 37 °C. At each time point, 1 mL of the solution was taken out and replaced with the same amount so that the parameters stayed the same. Fresh PBS was used to replace the sample volumes that were taken out. RP and CLV oil concentrations were determined at various time intervals. The analysis was carried out in triplicate, and the average and standard deviation of the results were given.

### 4.7 Antioxidant Activity

A 2,2-diphenyl-l-picrylhydrazil (DPPH) assay was used to determine the membrane’s free-radical scavenging capacity, and the antioxidant activity was measured spectrophotometrically. In this method, methanol solutions containing membranes at varying concentrations (0.1, 0.2, and 0.5 mg/mL) were prepared. Then, they were mixed with 3 mL of a methanol solution containing DPPH at a concentration of 0.1 mM. These mixtures were shaken and incubated at 37 °C for 4 h in the dark. Their absorbance was then measured at 517 nm with a spectrophotometer (BioTek SynergyHT). As a control, a DPPH methanol solution without a sample was used. The following formula (eq 2) was used to determine the DPPH scavenging effect:^23^
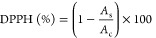
2where *A*_s_ represents the tested sample’s absorbance and *A*_c_ represents that of the control sample.

### 4.8 Statistical Analysis

The fiber diameter measurements of CA and CA blends with RP and CLV were statistically examined and presented as a mean and standard deviation (SD). A one-way analysis of variance (ANOVA) was performed to identify significant differences and then a Bonferroni post hoc test for multiple comparisons. All diameter measurements were obtained from SEM images using Image J (National Institute of Health, USA) at 50 repetitions for statistical analysis. Antibacterial and cell viability test results were presented as the mean ± standard deviation of each treatment. ANOVA was performed using SPSS 22.0 (SPSS Inc., Chicago, USA). The differences between means were evaluated by Tukey’s multiple range test (*p* < 0.05). The experiments were carried out in triplicate.
